# Protocol for generation and regeneration of PEG-passivated slides for single-molecule measurements

**DOI:** 10.1016/j.xpro.2022.101152

**Published:** 2022-02-03

**Authors:** Tapas Paul, Sua Myong

**Affiliations:** 1 Department of Biophysics, Johns Hopkins University, Baltimore, MD 21218, USA; 2 Physics Frontier Center, Center for the Physics of Living Cells, University of Illinois Urbana-Champaign, Urbana, IL 61801, USA

**Keywords:** Biophysics, Single-molecule Assays, Molecular Biology, Chemistry

## Abstract

Single-molecule fluorescence detection by total internal reflection microscope requires surface passivation by polyethylene glycol (PEG) coating, which is labor intensive and is only good for one or two experiments. Here, we present an efficient and reliable protocol for generating and regenerating the PEG surface for multiple rounds of experiments (∼5–10 times) in the same channel. This protocol is very simple, robust, rapid, and versatile; i.e., multiple strategies can be implemented to regenerate different layers of surface. The regeneration strategy saves time, improves the cost effectiveness, and enhances the efficiency of single-molecule experiments.

For complete details on the use and execution of this profile, please refer to Paul et al. (2021a).

## Highlights

• Regeneration of PEG-passivated slide is simple, quick, and cost effective
• Multiple experiments can be performed in a single channel
• Different strategies are implemented for different level of regeneration
• Regeneration leads to highly reproducible results

## Graphical abstract

**Figure:**
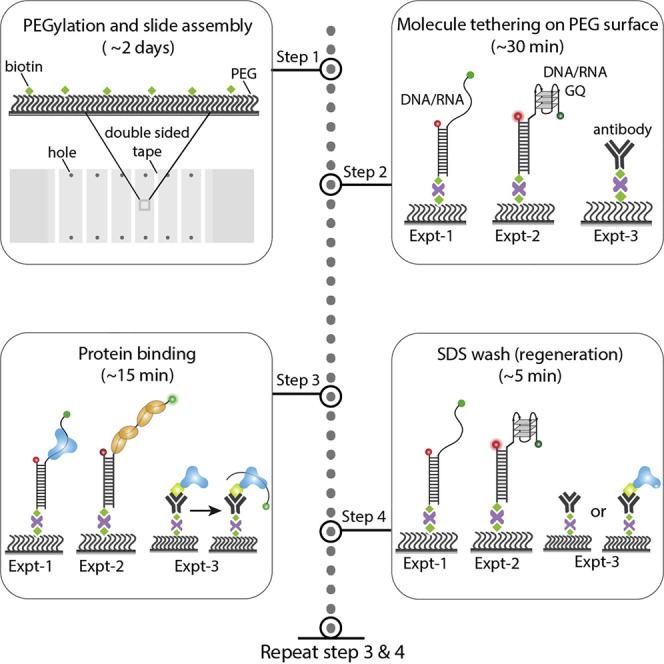

## Before you begin

This protocol describes how to generate the PEG-passivation on slides and reuse the same PEG-passivated channel in single-molecule measurements, especially in the context of DNA or RNA binding proteins and antibody regeneration during pull-down experiments. The workflow described in this protocol takes a few hours in addition to the time needed for surface passivation and preparation of buffer, samples, and calibration of the instrument (see materials and equipment). It is advisable that you perform the following steps:

First, prepare the required DNA or RNA and purify the protein-of-interest. Usually biotinylated and/or fluorescently labeled DNA or RNA molecules (from Integrated DNA Technologies) are tethered on the surface for single-molecule measurements. An untagged protein may be used when RNA or DNA are tethered to the surface.

Second, a tagged protein is required for antibody immobilization to the single-molecule surface during pull-down experiment.

Third, the total internal reflection fluorescence (TIRF) microscopy should be properly calibrated using a standard sample. Follow the safety protection protocol for lasers as per the institution’s recommendations.

### Preparing reagents and DNA or RNA annealing

**Timing: ∼1–2 h**

1. DNA or RNA annealing
  a. Mix the appropriate pairs of DNA or RNA strands in a 1:1.2 (biotin : non-biotin strand) molar ratio (final concentration 10 μM) in T50 or respective buffers.
  b. Anneal by incubating the solution at 95°C for 2 min, then gradually cool at the rate of 2 °C/min down to 40°C followed by 5°C/min cooling to reach 4°C using thermal cycler.
  c. Anneal stock stored at −20°C. For the G-quadruplex sample, anneal immediately before use.

### Slide preparation

**Timing: ∼2 days**

**Figure 1:**
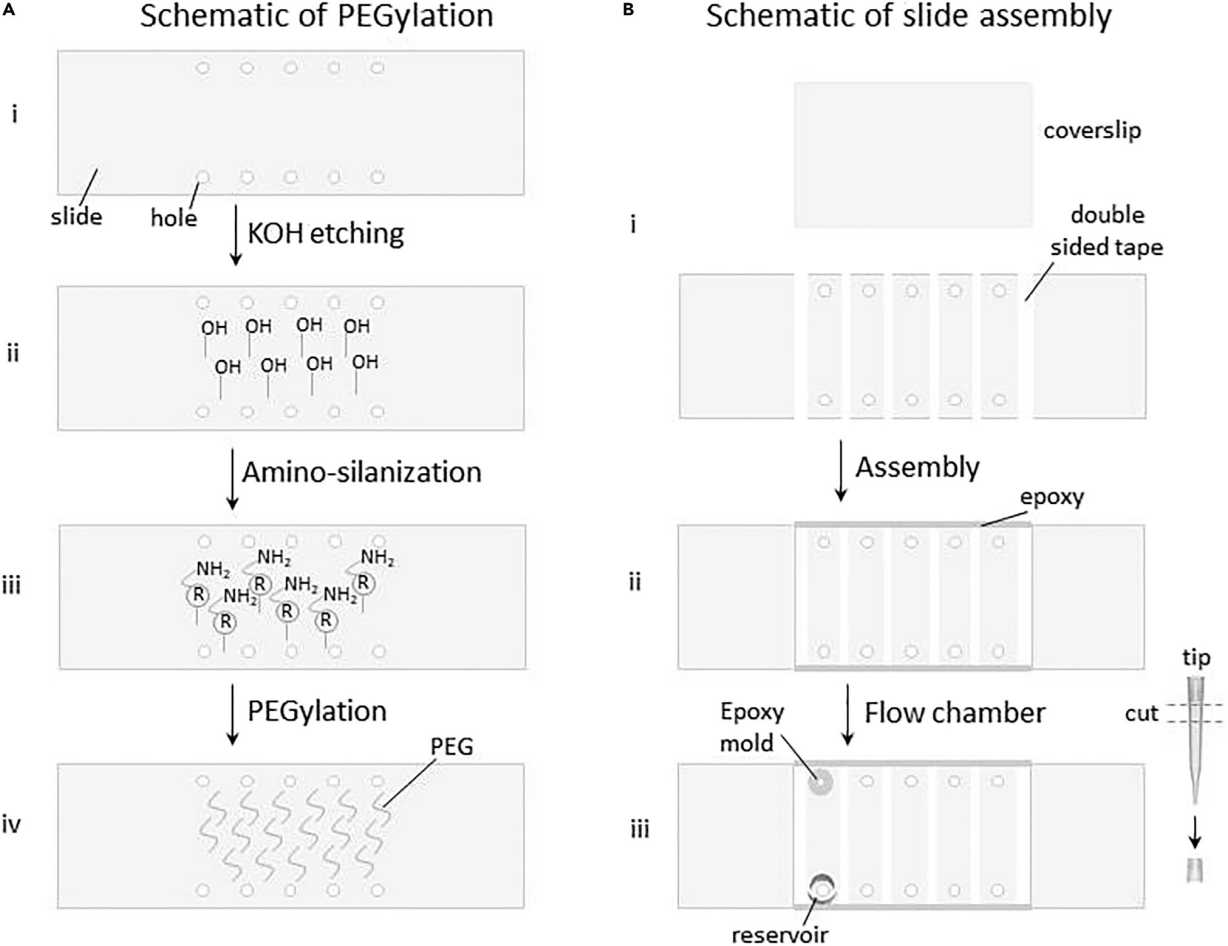
Schematic of the PEGylation protocol and preparation of sample chamber for smFRET experiment (A) Stepwise representation of surface passivation. (i) The slide with hole is properly cleaned. (ii) KOH treatment introduces the hydroxyl group (-OH) on the slide surface. (iii) The slide is ready for amino-silanization followed by PEGylation (iv). (B) Stepwise representation of slide assembly. (i) Slice of a double sided tape applied to the PEGylated slide surface in-between each holes. (ii) Sandwich the coverslip on the taped slide where PEGylated surface facing each other. Seal the tape with pipet tip and applying quick-drying epoxy at the two edges. (iii) Sample reservoir made with a cut piece of the pipet tip (beside contain) in one hole and epoxy mold created in other hole for the syringe with tube attachment.

The slide preparation protocol involves the following steps below; initial slide cleaning, KOH treatment, Amino-silanization and PEGylation. The walkthrough of slide preparation is shown in Figure 1.

2. Initial slide cleaning (slide size: 3” x 1”, 1 mm thick)
  a. For used slides, soak the slides ∼5-h in ethanol or acetone.
  b. Boil in water and sonicate (Ultrasonic bath water sonicator from Branson) in 5% (w/v) alconox detergent for 5–10 min.
  c. Wash with tap water and gently scrub the slide with a gloved finger.
  d. Wash with Milli-Q water and put the slides in a slide holder jar filled with Milli-Q water.
    ***Note:*** If start with new slides, drill (using diamond drilling needle) five or six pairs of holes side by side to make sure that they are covered with a coverslip. Then wash with water and scrub with a gloved finger. Afterward, put the slides in a slide holder jar and fill with Milli-Q water.
  e. Thoroughly burn (on propane torch burner) both side of the slides (using forcep to hold slides) to remove any trace particles. Cool the slides by blowing nitrogen gas onto the surface and put the slide into a dry slide holder jar.
    **CRITICAL:** Super-hot slides can overheat the slide holder jar and may crack it.
  f. Scrub the coverslips with a gloved finger, and place them in a water-filled slide jar (2–3 extra coverslips should be prepared as a backup).
    **CRITICAL:** Slides and coverslips should be handled with a clean pair of forceps for this entire protocol.
3. 1 M KOH treatment (Caution: strong base)
  ***Note:*** During the KOH treatment, equilibrate the aminosilane (from −20°C) at ∼25°C in drawer (aminosilane is photosensitive) for at least one hour to save time as well as pre-warm the chemicals to avoid the hydrolysis of aminosilane.
  a. Fill both the slide and coverslip holder jars with 1 M KOH.
  b. Sonicate (Ultrasonic bath water sonicator from Branson) the jars for 30–45 min.
  c. Discard KOH, rinse both the jars with Milli-Q water and then fill with Milli-Q water.
  d. Sonicate again for 20–30 min and then rinse with Milli-Q water.
  e. Burn the slides properly to dry them and cool them by blowing nitrogen gas and put another dry jar.
  f. Dry coverslips with nitrogen gas and burn 5–6 times with quick passes through the flame and put another dry jar.
    **CRITICAL:** Coverslips may break if burned for too long or with an intense flame.
  g. Rinse both jars with methanol and dry completely. Transfer all slides and coverslips into their respective jars.
    **CRITICAL:** Both jars should be dried completely as aminosilane hydrolyzes quickly in water.

4. Amino-silanization
  a. Fill 150 mL of methanol into a 500-mL properly cleaned and dry flask. Add 2 mL glacial acetic acid with 2 mL N-(2-Aminoethyl)-3-Aminopropyltrimethoxysilane.
  b. Mix the solution by shaking and swirling immediately and pour this buffer into the jars with slides and coverslips until it is full.
  c. Incubate in the dark for 10 min, sonicate for 1 min, and again incubate in the dark for 30 min.

***Note:*** During this incubation period, prepare a plastic box as a humid chamber for PEGylation.

5. PEGylation
  a. Prepare fresh 100 mM bicarbonate buffer (84 mg NaHCO_3_ in 10 mL water).
  b. Rinse both slides and coverslips with a generous amount of Milli-Q water and then dry by flowing nitrogen gas.
    **CRITICAL:** Take extra care otherwise coverslips may break during this wash due to the water flow and nitrogen stream.
  c. Weigh out 180–190 mg methoxy poly(ethylene glycol) succinimidyl valerate (mPEG-SVA) and ∼2–3 mg Biotin-PEG-SVA per 12 slides and add to 900 μL bicarbonate buffer.
  d. Gently pipette up and down to mix well.
  e. Centrifuge the PEG/Biotin-PEG solution at 12,000×*g* for 1 min (solution should be clear).
  f. Put 70 μL of PEG buffer onto each slide and sandwich it immediately with the coverslip and make sure not to include air bubbles during this process.
    ***Note:*** Carefully lay the coverslips on the slides using a forcep with sharp/fine tips to avoid air bubbles inside.
    ***Note:*** The half-life of succinimidyl valerate PEG is only ∼10 min. After adding the bicarbonate buffer to PEG, immediately apply to slides.
  g. Incubate in the dark at least ∼3–5 h.
    ***Note:*** Two rounds of PEGylation can remarkably improve the passivation quality. For some critical experiments like pull-down, double PEGylation is recommended (repeat step 5).

6. Storage of PEG slides
  a. Wash both slides and coverslips with a generous amount of Milli-Q water again.
  b. Dry the slides and coverslips with the nitrogen gas stream.
  c. Carefully transfer each slide/coverslip pair to a 50-mL tube. The PEGylated slides should face away from each other.
  d. Vacuum-seal with a food saver bag and store them at −20°C.

## Key resources table

**Table:** 

REAGENT or RESOURCE	SOURCE	IDENTIFIER
**Antibodies**		

GFP Antibody Biotin Conjugated (Rabbit) (Dilute to use ∼10 nM or 500–1000× times)	Rockland Antibodies & Assays	Cat#600406215
Histidine Antibody Biotin Conjugated (Dilute to use ∼10 nM or 500–1000× times)	QIAGEN	Cat#34660
MBP Antibody Biotin Conjugated (Dilute to use ∼10 nM or 500–1000× times)	Rockland Antibodies & Assays	Cat#200406385S

**Chemicals peptide, and recombinant proteins**		

Alconox™ Powdered Precision Cleaner	Fisher Scientific	Cat#16000104
Biotin	Sigma-Aldrich	Cat#2003993
BSA	New England BioLabs	Cat#B9000S
GdmCl	Sigma-Aldrich	Cat#200-002-3
Urea	Sigma-Aldrich	Cat#200-315-5
Proteinase-K	New England BioLabs	Cat#P8107S
SDS	Fisher Scientific	Cat#BP166-100
NaOH	Sigma-Aldrich	Cat#1310-73-2
Yeast t-RNA	Thermo Scientific	Cat#AM7119
Biotin-PEG-SVA, MW 5,000	Laysan Bio Inc.	Cat#Biotin-PEG-SVA-5000-100mg
Catalase from bovine liver	Sigma-Aldrich	Cat#C3155
Glucose Oxidase from Aspergillus niger	Sigma-Aldrich	Cat#G2133
mPEG-Succinimidyl Valerate, MW 5,000	Laysan Bio Inc.	Cat#MPEG-SVA-5000-1g
N-(2-Aminoethyl)-3-Aminopropyltrimethoxysilane	United Chemical Technologies	Cat#1760-24-3
NeutrAvidin Protein	Thermo Scientific	Cat#31000
Trolox(R), 97%, ACROS Organics™	Fisher Scientific	Cat#AC218940010

**Oligonucleotides**		

Biotin-18merRNA: 5’-/biotin/rUrGrG rCrGrA rCrGrG rCrArG rCrGrA rGrGrC/Cy5/ -3′	Integrated DNA Technologies	N/A
U50-18mer: 5’-/Cy3/rUrUrU rUrUrU rUrUrU rUrUrU rUrUrU rUrUrU rUrUrU rUrUrU rUrUrU rUrUrU rUrUrU rUrUrU rUrUrU rUrUrU rUrUrU rUrUrU rUrUrG rCrCrU rCrGrC rUrGrC rCrGrU rCrGrC rCrA -3′	Integrated DNA Technologies	N/A
Biotin-18merDNA: 5’-/Cy5/GCC TCG CTG CCG TCG CCA/Biotin/-3′	Integrated DNA Technologies	N/A
18mer-G4-T9: 5′-TGG CGA CGG CAG CGA GGC TT GGG T GGG T GGG TA GGG TTT TTT TTT/Cy3/-3′	Integrated DNA Technologies	N/A

**Software and algorithms**		

IDL	Harris Geospatial	https://www.l3harrisgeospatial.com/Software-Technology/IDL
MATLAB	MathWorks	https://www.mathworks.com/products/matlab.html
smCamera	(Roy et al., 2008)	https://cplc.illinois.edu/research/tools

**Other**		

5 Minute® Rapid-Curing, General Purpose Adhesive Epoxy, 25 mL Tube	All-Spec	Cat#14250
Chemyx Inc fusion 200 Touch Syringe Pump	Fisher Scientific	Cat#NC0670590
ETT PTFE Tubing Size 26, 100 ft, Natural Color	Weico Wire	Cat#ETT-26
Quartz Microscope Slides	G Finkenbeiner Inc.	N/A
Scotch® 665 Permanent Double-Sided Tape, 1/2" x 250", Clear, Pack Of 3 Rolls	Office Depot	Cat#391775
TetraSpeck™ Microspheres, 0.1 μm, fluorescent blue/green/orange/dark red	Thermo Fisher Scientific	Cat#T7279
TIRF Microscope	Olympus	N/A
532 and 634-nm diode laser	Compass 315M; Coherent, Santa Clara, CA	N/A

## Materials and equipment

A prism-type total internal reflection fluorescence (TIRF) microscope equipped with a solid-state 532- and 634-nm diode laser is required for these single-molecule measurements. Many of these scopes are homebuilt, and there are papers with detailed instructions for constructing and optimizing TIRF microscopes (Joo and Ha, 2012). Because of the complexity of building TIRF microscopes, we refer the reader to these publications for further instructions (Paul et al., 2016; Roy et al., 2008). A syringe pump is required for real-time flow experiments wherein buffer/sample solutions are pulled from the reservoir with a 1 mL syringe (Yang and Ha, 2018).

Prepare 10% SDS, 8 M Urea, 8 M GdmCl and 10 M NaOH stock in Milli-Q water and filter through the 0.22-*μ*m membrane filter.

**Table:** 10× T50 Buffer (10 mL)

Reagent	Final concentration (mM)	Amount
1 M Tris, pH 7.4	100 mM	1 mL
5 M NaCl	500 mM	1 mL
Milli-Q H_2_O	n/a	8 mL
**Total**	**n/a**	**10 mL**

**Table:** 10× TK100 Buffer (10 mL)

Reagent	Final concentration (mM)	Amount
1 M Tris, pH 7.4	100 mM	1 mL
4 M KCl	1000 mM	2.5 mL
Milli-Q H_2_O	n/a	6.5 mL
**Total**	**n/a**	**10 mL**

**Table:** 10× TN100 Buffer (10 mL)

Reagent	Final concentration (mM)	Amount
1 M Tris, pH 7.4	100 mM	1 mL
5 M NaCl	1000 mM	2 mL
Milli-Q H_2_O	n/a	7 mL
**Total**	**n/a**	**10 mL**

**Table:** 10× TMg5 Buffer (10 mL)

Reagent	Final concentration (mM)	Amount
1 M Tris, pH 7.4	100 mM	1 mL
2 M MgCl_2_	50 mM	0.25 mL
Milli-Q H_2_O	n/a	8.75 mL
**Total**	**n/a**	**10 mL**

**Table:** 10× helicase Buffer (10 mL)

Reagent	Final concentration (mM)	Amount
1 M Tris, pH 7.4	100 mM	1 mL
4 M KCl	1000 mM	2.5 mL
Milli-Q H_2_O	10 mM	0.05 mL
dH_2_O	n/a	6.45 mL
**Total**	**n/a**	**10 mL**

**Table:** 100× Gluoxy (100 μL)

Reagent	Final concentration (mM or μM)	Amount
Glucose Oxidase	n/a	10 mg
Catalase	n/a	2 μL
T50 Buffer	n/a	98 μL
**Total**	**n/a**	**100 μL**

**Table:** Imaging Buffer (500 μL)

Reagent	Final concentration (mM or μM)	Amount
10× experimental buffer	1× buffer	10 μL
20% (w/v) glucose	0.4% (w/v)	1 μL
100× Gluoxy	1×	1 μL
10 mM Trolox	∼10 mM	88 μL
**Total**	**n/a**	**100 μL**

***Note:*** All the 10 X buffer should be filtered through the 0.22-*μ*m membrane filter. Prepare fresh imaging buffer just before the experiment. Gluoxy can be stored at 4°C for up to 8 weeks. Trolox (10 mM) can be stored at −20°C for up to 6 months and at 4°C for up to 8 weeks.

***Alternatives:*** Protocatechuic acid-protocatechuate-3,4-dioxygenase (PCA-PCD) can be used instead of glucose oxidase and glucose.

## Step-by-step method details

### Slide assembly

**Timing: ∼1 h**

The single-molecule slide is assembled by attaching the coverslip and slide with double sided tape and sealed two edges with epoxy. For flow experiments, epoxy molds and buffer reservoirs are added for automated flow of solutions. The walkthrough of slide assembly is shown in Figure 1.

***Note:*** Take out the stored slides tube from −20°C freezer and equilibrate them at ∼25°C for ∼15–20 min.

1. Make microfluidic channel
  a. Tear off ∼5–7 cm long piece of double-sided tape (from Scotch brand of 0.1 mm thickness) and paste on a clean glass surface. Slice the tape into ∼1 mm-wide strips using a clean razor blade.
  b. Carefully take out the slide from the tube and place the PEGylated side face-up on a clean surface.
  c. Take out the coverslip and place the PEGylated side face-up onto the top of the tip box.
  d. Lift the tape strip (from step a) using the razor blade and gently apply it in-between each hole to the PEGylated slide surface.
  e. Place the coverslip on the taped slide. The PEGylated side of the slide and coverslip should be facing each other.
  f. Gently press the coverslip with a 200-μL pipet tip (or 1000- μL pipet tip and/or the blunt edge of a razor blade) to properly seal the tape.
  g. The edges of the slide-coverslip sandwich are sealed by applying quick-drying epoxy with a 10-μL pipet tip.
    ***Note:*** Excessive epoxy can block the holes by flowing in via capillary action.
  h. Wait ∼5–10 min for complete drying of the epoxy.

2. Make the reservoir for real-time flow experiments
  a. Cut the upper part of 200 μL pipet tips into small pieces that can be used for buffer reservoir.
  b. Using epoxy, the buffer reservoir is attached around each of the drilled holes at the top of the slide.
  c. Insert 10 μL pipet tips into the other drilled holes and seal with epoxy to create a mold for the syringe attachment.
  d. Wait ∼15–20 min until the epoxy has completely dried.
  e. Carefully remove the 10 μL pipet tips.
  f. Cut the 10 μL pipet tip and use the lower part to attach 2 mm tube inside the cut tip using epoxy.
  g. Attach the tube to a 1 mL syringe with a needle Luer-lock.

### Instrument calibration

**Timing: ∼30 min**

The TIRF instrument should be calibrated before doing real-time single-molecule measurements.

3. Prepare the standard bead slide
  a. Dissolve 1 μL tetraspeck standard fluorescent beads in 99 μL T50 buffer.
  b. Vortex for 1 min three times.
  c. Pipet 50 μL of diluted low-density bead solution onto the premade flow chamber of a non-PEGylated quartz/glass slide.
  d. Wait for 3–5 min and then wash with T50 buffer containing 0.1 N HCl and ∼5% glycerol.
    ***Note:*** To adjust the molecular density, observe the bead slide using the TIRF microscope before sealing the slide. If there are too few beads, an additional bead solution can be added. If there are too many beads, discard the channel and apply a more dilute solution to another channel. The optimum number of beads is ∼300–400 spots per imaging area of 2,500 μm^2^.
  e. Seal the channel with fast-drying epoxy and ensure that there are no air bubbles inside.
  f. Allow the epoxy to solidify for ∼10 min. Transfer to a 50 mL conical vial for safekeeping and store at 4°C for further use.

4. Map the donor/acceptor channel using a standard bead slide (instrument calibration).
  a. Carefully apply one drop (∼80 μL) of Milli-Q water to the objective on the TIRF microscope.
  b. Gently place the slide on the microscope; the coverslip should be facing down toward the objective.
  c. Properly clean the prism with ethanol using soft Kimwipes or lens cleaning tissue. Add ∼1 drop of immersion oil and fix the prism to the microscope so that the oil surface is touching the slide.
  d. Turn on the fluorescent laser to illuminate the beads. Adjust the microscopic stage as needed to properly focus on the fluorescent beads.
  e. Optimize the data scaler, background, gain, exposure time, and laser intensity as needed.
  f. Record ∼2 s short movies and follow the quantification steps below for mapping. The mapping efficiency should be at least ∼85%. (see quantification and statistical analysis for further instructions).

### Method 1: 0.1% SDS to efficiently remove the DNA- or RNA-bound protein

**Timing: ∼ 1–2 h**

This step uses FRET-labeled DNA or RNA constructs, which can be tethered on a PEGylated slide via biotin-NeutrAvidin linkage. The tagged or untagged protein-of-interest can be flown onto the slide for experiments. This protocol uses 0.1% SDS, which is highly effective for repeatedly regenerating the single-molecule surface by removing all the DNA or RNA-bound protein. This process restores the clean, unbound substrate tethered to the surface.

5. Regeneration of surface-tethered DNA or RNA-bound protein.
  a. Put the assembled slide (from step 1, Slide Assembly) on the TIRF microscope (follow step 4, Instrument Calibration). (Troubleshooting 1)
  b. Flow 50 μL NeutrAvidin (100 μg/mL) in either T50 or experimental buffer into the sample chamber of the slide.
  c. Incubate for ∼1–2 min.
  d. Wash the sample chamber with 50 μL T50 or experimental buffer.
  e. Flow 50 μL of 20 pM biotinylated and FRET-labeled DNA or RNA construct.
  f. Incubate for ∼1–2 min.
  g. Wash the sample chamber with 50 μL experimental buffer.
  h. Flow 50 μL imaging buffer.
  i. Turn on the laser for movie recording. The microscope stage needs to be adjusted for proper focusing of the single-molecule surface.
    ***Note:*** The DNA or RNA density on the single-molecule surface should be approximately 300–400 molecules per field of view. (Troubleshooting 2)
  j. Record 20 short movies (2 s) at different imaging areas with the donor excitation laser on for making the FRET histogram. A shutter manager can be used to alternatively excite the donor and acceptor dyes.
  k. Record 3–4 long movies (120 s) at different imaging areas with the donor excitation laser to record the individual molecular behavior.
  l. Flow 50 μL of imaging buffer with your protein-of-interest at the desired concentration.
    ***Note:*** If the flow assembly setup with the reservoir is being used, acquire a long video (180 s) while flowing the protein at the 10 s mark into the sample chamber with the syringe pump.
    ***Note:*** For all single-molecule flow measurements using the syringe pump, it is highly recommended that less volume is withdrawn than is added to the buffer reservoir. For instance, if 50 μL of the solution is added to the reservoir, 40 μL should be withdrawn by the pump to make sure that the slide chamber is not empty. A slower flow rate of ∼1 mL/min is recommended.
  m. Follow steps 5j-k again to record the protein-bound movies before and after buffer wash (to remove free protein).
    ***Note:*** Depending on the experimental condition, more movies can be recorded after protein incubation.
    ***Note:*** Make sure no air bubbles pass through the channel while washing off the free protein.
  n. Flow 50 μL of 0.1% SDS in T50 or experimental buffer.
    **CRITICAL:** Maintain the salt concentration during SDS wash to stabilize the DNA or RNA. T50 or 100 mM NaCl or 1–5 mM MgCl_2_ salt-containing buffer is recommended during SDS wash. Avoid KCl during the SDS wash because it precipitates to SDS-potassium complex, which reduces the SDS effect in denaturing the protein.
    ***Note:*** Use freshly prepared SDS. Old SDS stock reduces the effectiveness of regeneration.
  o. Incubate for ∼1 min.
  p. Wash 2–3 times with 50 μL T50 or experimental buffer.
  q. Flow 50 μL imaging buffer.
  r. Follow steps 5i-m. This is the first round of regeneration.
  s. Follow steps 5n-q and then steps 5i-m for the second round of regeneration.
  t. Continue it for multiple rounds of regeneration (efficiently regenerate at least for 10 times).
  u. The recorded movies are processed and analyzed as described below (see quantification and statistical analysis).
    ***Note:*** Other protein denaturants may be used during regeneration (e.g., 6 M Urea, 6 M GdmCl or 100 μM Proteinase-K), but the regeneration efficiency of 0.1% SDS is much higher compared to all others.

### Method 2: Duplex substrate regeneration

**Timing: ∼1 h and more**

This method details two different strategies to regenerate the duplex DNA or RNA substrate. First, we describe helicase-induced duplex substrate regeneration, which requires a helicase that unwinds the DNA or RNA substrate; the FRET-labeled DNA or RNA construct is dissociated by a helicase treatment, leaving the biotinylated strand on the surface for annealing to a new DNA or RNA strand. (Paul et al., 2020). This unwinding leads to the loss of FRET pair, which can be visualized by laser excitation. Alternatively, one can use 50 mM NaOH, which also dissociates the duplexed RNA or DNA. Again, the biotinylated single-stranded DNA or RNA remains bound to the surface while the non-biotinylated strand is washed away. In both cases, the complementary ssDNA or ssRNA is flowed to regenerate a new RNA or DNA duplex.

***Note:*** Using this regeneration process, one duplex can be exchanged with another if the applied ssDNA or ssRNA is complementary to the surface-tethered ssDNA or ssRNA.

6. Helicase-induced duplex substrate regeneration (any DNA or RNA unwinding helicase).
  a. Follow steps 5a-k to immobilize the DNA or RNA construct for helicase experiments.
  b. Flow 50 μL imaging buffer with your helicase-of-interest at the desired concentration and conditions.
  c. Record the movies until the unwinding process is completed.
    ***Note:*** Helicase-induced duplex unwinding leaves behind the biotinylated single-strand DNA or RNA.
  d. Flow 50 μL of 5 nM complementary ssDNA with 5 mM MgCl_2_ containing buffer. (Troubleshooting 3)
  e. Incubate for ∼5 min.
  f. Wash 2–3 times with 50 μL of experimental buffer.
  g. Flow 50 μL imaging buffer and record movies.
  h. Repeat the unwinding process by following steps 6b-c.
  i. Continue regenerating the process if desired by following steps 6b-h.

7. NaOH-induced duplex substrate regeneration
  a. Follow steps from 5a-k to immobilize the DNA or RNA and record movies.
  b. Flow 50 μL of 50 mM NaOH.
  c. Incubate for ∼1 min.
  d. Wash 2–3 times with 50 μL T50 or experimental buffer.
    ***Note:*** NaOH denatures the surface-bound duplex and leaves behind the biotinylated, surface-tethered single-strand DNA or RNA.
  e. Flow 50 μL of 5 nM complementary ssDNA with 5 mM MgCl_2_ containing buffer. (Troubleshooting 3)
  f. Incubate for ∼5 min.
  g. Wash 2–3 times with 50 μL buffer.
  h. Flow 50 μL imaging buffer and record movies.
  i. If desired, repeat the regeneration and data acquisition protocol from 7b-h.

### Method 3: Breaking the biotin-streptavidin/NeutrAvidin linkage

**Timing: ∼2 h**

This method describes the breakage of the biotin-NeutrAvidin linkage using 7 M NaOH. The PEG-biotin is revealed by stripping the NeutrAvidin layer. Therefore, this protocol regenerates the PEG-biotin surface, which can then be reused for entirely different experiments.

8. 7 M NaOH (Caution: strong base) breaks the biotin-NeutrAvidin linkage.
  a. Follow steps from 5a-k to immobilize the DNA or RNA construct and record movies.
  b. Flow 50 μL of 7 M NaOH.
  c. Incubate for ∼2–3 min.
  d. Wash 2–3 times with 50 μL T50 or experimental buffer.
  e. Follow steps from 5b-t for DNA or RNA immobilization and continue regeneration using 0.1% SDS, if desired.

The sample can be changed by repeating the regeneration process. Follow steps 8b-e as needed. (Troubleshooting 3 and 4)

### Method 4: Antibody regeneration

**Timing: ∼2 h**

This method describes the single-molecule pull-down experiments where 0.1% SDS is used to regenerate the surface bound antibody. Generally, in pull-down assay, biotinylated antibodies against the protein-of-interest are immobilized on a passivated microscope slide. The surface-tethered antibody captures the partner protein through physiological interaction (Jain et al., 2011). Then, applied non-biotinylated fluorophore label DNA/RNA interact with immobilized protein and the fluorescence signal is detected through TIRF microscopy.

9. Regeneration of antibody using 0.1% SDS.
  a. Follow steps from 5a-d.
  b. Flow 50 μL of 10 nM biotin conjugated antibody either in T50 or experimental buffer.
    ***Note:*** In pull down experiments, the exact antibody used is dependent on the tag attached on the corresponding protein-of-interest. For instance, biotinylated anti-GFP antibodies (Rockland Antibodies & Assays, Cat#600406215) are used for GFP-tagged protein, and anti-His antibodies (Qiagen, Cat#34660) are used for His-tagged proteins. The antibody dilution (∼10 nM) needs to be optimized for each protein-antibody pair (generally in the range of 100× to 2000× dilution). (Troubleshooting 5)
  c. Incubate for 3–5 min.
  d. Wash with 50 μL T50 or experimental buffer.
  e. Flow 50 μL 10 nM tagged protein (corresponding to the surface-tethered antibody) in T50 or experimental buffer.
  f. Incubate for ∼10 min.
  g. Wash with 50 μL T50 or experimental buffer.
    ***Note:*** Make sure no air bubbles pass through the channel during the entire pull-down experiment.
  h. Flow 50 μL of 10 nM non-biotinylated DNA or RNA construct that can bind with surface-tethered protein. (Troubleshooting 3)
  i. Incubate for ∼10 min.
  j. Wash with 50 μL of T50 or experimental buffer.
  k. Flow 50 μL of imaging buffer.
  l. Turn on the excitation laser for recording movies. The microscope stage needs to be adjusted for proper focusing of the single-molecule surface.
    ***Note:*** The DNA or RNA density on the single-molecule surface needs to be optimized approximately 300–400 molecules per field of view.
  m. Record 20 short movies (2 s) at different imaging areas with the donor excitation laser for making the FRET histogram, if applicable. A shutter manager can be used to excite the donor and acceptor dyes.
  n. Record 3–4 long movies (120 s) at different imaging areas with the donor excitation laser to visualize individual molecules.
  o. Flow 50 μL of 0.1% SDS with T50 or experimental buffer.
  p. Incubate for ∼1 min.
  q. Wash 2–3 times with 50 μL of T50 or experimental buffer.
  r. Follow steps 9h-n to perform additional experimental conditions. This is one round of regeneration.
    ***Note:*** Treatment with 0.1% SDS is usually not harsh enough to disrupt the antibody interactions of surface-bound protein, but it is sufficient to release the bound DNA or RNA. If 0.1% SDS is sufficient to disrupt the antibody-protein interaction, repeat steps 9e-n to flow the protein-of-interest again.
  s. Follow steps 9o-r for another round of regeneration and repeat for multiple rounds of regeneration as needed.
    ***Note:*** Use the 7M NaOH treatment to change one biotinylated antibody to another biotinylated antibody following the steps described in Method 3.

## Expected outcomes

**Figure 2:**
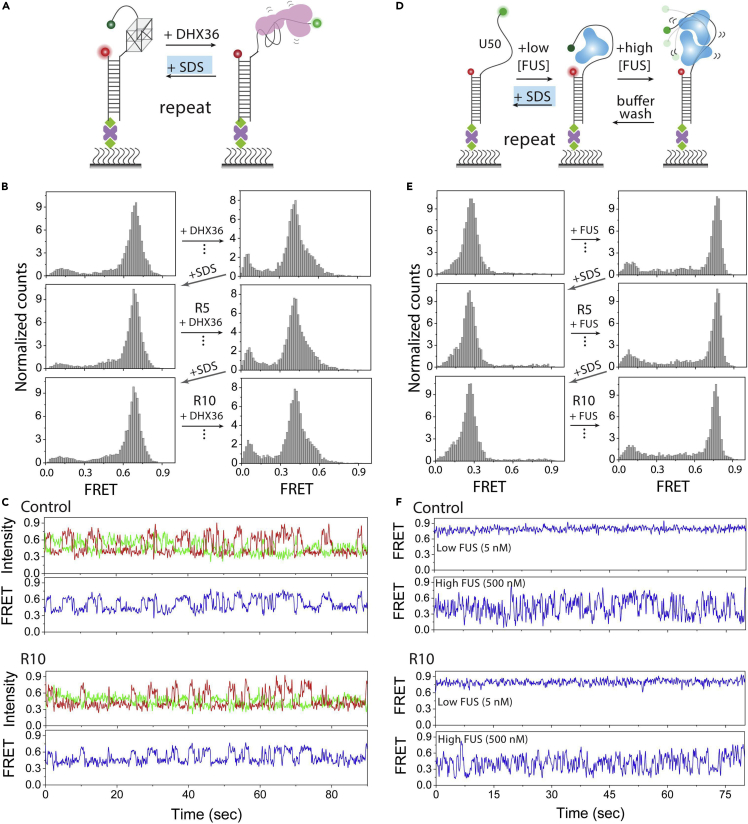
Example of DNA/RNA regeneration on PEG surface by 0.1% SDS treatment (A and D) Schematic smFRET experimental regeneration model of (A) G4 with T9 tail and DHX36 binding, and (D) 50 poly U50 tail and FUS engagement. (B and E) The FRET histogram of control, 5^th^ (R5) and 10^th^ (R10) repeats of respective protein binding. (C and F) Representative smFRET traces of control and 10^th^ (R10) repeats of regeneration of (C) DHX36 bound to G4, (F) FUS bound to poly U50.

Here, we describe the protocol for preparing PEG-passivated slides for TIRF microscopy and four possible strategies to regenerate the single-molecule surface for multiple experiments. Three chemical solutions (0.1% SDS, 50 mM NaOH, and 7 M NaOH) are used to regenerate the PEG surface in a highly reliable manner. We regenerated the PEG surface using our previous experimental models with highly reproducible results (Lee et al., 2020; Niaki et al., 2020; Tippana et al., 2019; Paul et al., 2021b). A typical example of reproducible histograms and consistent single-molecule traces following regeneration are shown in Figure 2. The results are highly reproducible for each regeneration step, and these are very simple and rapid strategies that will enhance the efficiency of single-molecule experiments. All the regeneration experimental strategy depends on the experimental design. Below are the expected outcomes for each of the four methods discussed above:

Method 1 (0.1% SDS efficiently remove the DNA or RNA bound protein): The surface-tethered DNA or RNA FRET constructs are regenerated within ∼1–2 min by washing out the bound protein using 0.1% SDS. At least 10 consecutive experiments in different DNA-/RNA-protein systems produce indistinguishable results both in molecule count and protein activity.

Method 2 (duplex substrate regeneration): Duplexed DNA is unwound by helicase or denatured by 50 mM NaOH which leaving behind the biotinylated single-stranded DNA or RNA. A new complementary strand is reannealed to either recover the same duplex or convert to a different substrate. Nearly 10 rounds of regeneration can perfectly recover the substrate in terms of molecule count and protein activity.

Method 3 (Breaking the biotin-streptavidin/NeutrAvidin linkage): NaOH (7 M) strips the NeutrAvidin from the biotin-PEG layer and the PEG-biotin surface is fully regenerated by coating with fresh NeutrAvidin five times in a row. Using the 7 M NaOH regeneration strategy combined with 0.1% SDS, a series of five different experiments involving DNA, RNA, and proteins in the same channel is possible depending on the experimental needs.

Method 4 (Antibody regeneration): Surface tethered biotin tagged antibody is successfully regenerated (for both 0.1% SDS and 7 M NaOH) at least for five times.

## Quantification and statistical analysis

Data can be visualized and analyzed using any program of choice. Analysis of nearly all single-molecule measurements follows the same initial flow. First, individual molecules are mapped (see instrument calibration). Second, recorded single-molecule movies are processed using a standard mapping file on IDL (see data and code availability). Third, the processed data are used to build histograms, and the individual traces are analyzed using MATLAB code (see data and code availability). The data analysis for each round of regeneration will follow the same general pathway. Though the regeneration strategy reproduces the same results, we still recommend replicating the experiments in separate trials.

Statistical comparison of single-molecule measurements as well as reproducible results during regenerations is desirable. We recommend testing with a normal distribution to choose the proper statistical test. Usually, an unpaired t-test should be used for pairwise comparison of two conditions, and it also can be used to compare the regeneration data.

## Limitations

We describe the regeneration technique of single-molecule FRET measurements, but the lack of specialized equipment like prism-type TIRF microscopes can limit their broad use among the scientific community. These regeneration strategies require the covalent linkage of passivated PEG polymer to the slide surface. Other passivation methods of non-covalently blocking the surface with BSA-biotin are not suitable for chemical treatment during regeneration. Each step during PEG passivation requires extra care to reduce nonspecific binding during regeneration. We suggest 0.1% SDS for regeneration but a higher concentration of SDS may be required for highly stable protein. Other denaturing chemicals like 6 M urea, 6 M GdmCl, or 100 μM Proteinase-K are not sufficient for this regeneration. During duplex annealing and after 7 M NaOH treatment, the surface needs to be blocked with biotin (1 μM), BSA (0.4 mg/mL), and yeast t-RNA (0.2 mg/mL) to avoid the nonspecific binding after 2–3 round of regeneration. Inadequate maintenance of experimental conditions in between regeneration might also compromise the results. If the regeneration results are not satisfactory, then the experiment should be stopped and a new channel should be employed.

## Troubleshooting

### Problem 1

TIRF spot is difficult to focus (Method 1, step 5).

### Potential solution

The fluorescence of bead slide is extremely bright and should make it easier to find the focus of TIRF spot by adjusting the stage and XYZ plane. This alignment helps the focusing process for single-molecule real experimental conditions. If it is still impossible to find the correct TIRF focus from experimental slides, then target the bright spot from scratch/junk on the slide surface and realign the laser by adjusting the stage and XYZ plane to find the TIRF spot. Sometimes evanescent field (i.e., single-molecule region) will not be clearly visible if the used oil in-between prism and slide gets deformed the surface. Hence, one should simply clean the prism/slide and reattempt the alignment to solve this issue.

### Problem 2

There are too many/too few bound molecules on the single-molecule surface (Method 1, step 5).

### Potential solution

Low concentration (∼20 pM) of biotin-labeled DNA or RNA should be incubated for ∼1–2 min to allow binding to the PEG-coated slide surface via NeutrAvidin interactions. If there are too few molecules, then flow the diluted DNA or RNA into the same channel again and incubate for ∼1–2 min to reach ∼300–400 molecules. If the optimal number of molecules still is not achieved, then check the NeutrAvidin activity or increase the DNA or RNA concentration. If too many molecules (>500) are bound to the slide surface, then follow the 7 M NaOH regeneration strategy and control the number of molecules as mentioned above.

### Problem 3

There is too much nonspecific binding during regeneration (Method 2, steps 6 and 7; Method 3, step 8; Method 4, step 9).

### Potential solution

Though PEG passivation greatly reduces the nonspecific binding of DNA/RNA/protein on the slide surface, nonspecific binding may occur during the regeneration protocol for Methods 2, 3, and 4. G4-containing substrates and proteins tend to nonspecifically stick to the surface. In this case, the surface can be blocked by applying biotin (1 mM), BSA (0.4 mg/mL), and yeast t-RNA (0.2 mg/ mL) with a ∼4–5 min incubation, which greatly reduces nonspecific binding.

### Problem 4

7 M NaOH treatment reduced the passivation quality (Method 3, step 8).

### Potential solution

7 M NaOH is a strong reagent, it may reduce the surface passivation quality after several applications. Therefore, it is recommended to block the surface with biotin (1 mM), BSA (0.4 mg/mL), and yeast t-RNA (0.2 mg/mL) after each regeneration as stated above. After 5 times of regeneration using 7 M NaOH, it is recommended not to use the same channel for further experiment.

### Problem 5

There are too many molecules during the pull-down experiment (Method 4, step 9).

### Potential solution

Non-biotinylated DNA or RNA substrates may bind with the surface-tethered protein during pull-down experiments. One can follow the same strategies in Problem 2 above to alter the surface labeling.

## Resource availability

### Lead contact

Further information and requests for resources and reagents should be directed to and will be fulfilled by the lead contact, Sua Myong (smyong@jhu.edu).

### Materials availability

This study did not generate new unique reagents.

## Data Availability

Single-molecule data acquisition and analysis package can all be obtained freely from CPLC’s website (https://cplc.illinois.edu/research/tools). MATLAB code from this manuscript can be downloaded from Github: (https://github.com/Myong-Lab). IDL (http://www.exelisvis.co.uk/ProductsServices/IDL.aspx), and MATLAB (https://www.mathworks.com/) can be downloaded with academic or individual licenses from their respective distributors.
